# Behavioral and Clinical Characteristics of American Indian/Alaska Native Adults in HIV Care — Medical Monitoring Project, United States, 2011–2015

**DOI:** 10.15585/mmwr.mm675152a1

**Published:** 2019-01-04

**Authors:** Amy R. Baugher, Linda Beer, Heather M. Bradley, Mary E. Evans, Qingwei Luo, R. Luke Shouse

**Affiliations:** ^1^Division of HIV/AIDS Prevention, National Center for HIV/AIDS, Viral Hepatitis, STD, and TB Prevention, CDC; ^2^Epidemic Intelligence Service, CDC; ^3^ICF International, Rockville, Maryland.

The rate of diagnosis of human immunodeficiency virus (HIV) infection among American Indians and Alaska Natives (AI/ANs) in 2016 (10.2 per 100,000 population) was the fourth highest among seven racial/ethnic groups in the United States (*1*); the number of diagnoses of HIV infection among AI/AN persons increased by 70%, from 143 in 2011 to 243 in 2016 (*1*). However, little has been published about the sociodemographic, behavioral, and clinical characteristics of AI/AN patients with HIV infection in care because small sample sizes have led to infrequent analysis of AI/AN-specific estimates (*2*) and because of underestimation of AI/AN race/ethnicity in surveillance and other data sources (*3*). CDC analyzed data from the Medical Monitoring Project (MMP), a surveillance system that collects information about the experiences and needs of persons with diagnosed HIV infection, collected during 2011–2015 among AI/AN adults receiving HIV medical care. The results indicated that 64% of AI/AN patients with HIV infection in care achieved sustained viral suppression, and 76% achieved viral suppression at their most recent viral load test within the past 12 months, which is below the national HIV prevention goal of 80%, but comparable to or better than some other racial/ethnic groups (*4*). Based on self-report, 51% of AI/AN patients with HIV infection had incomes at or below the U.S. Department of Health and Human Services’ (HHS) annual poverty limit, 27% had symptoms of depression, 78% reported internalized HIV-related stigma, and 20% reported binge drinking in the past 30 days. To improve the health of AI/AN patients with HIV infection, it is important that health care providers, tribal organizations, and state and local health departments consider the sociodemographic and behavioral barriers to AI/AN patients with HIV infection achieving viral suppression and design care plans that seek to eliminate those barriers.

MMP used a three-stage sample design (states and territories, facilities, patients). Response rates were 100% (states and territories), 83%–85% (range across cycles for facilities), and 49%–55% (patients). Data were collected using face-to-face or telephone interviews and medical record abstraction during June 2011–May 2015. Data were weighted for unequal selection probabilities and nonresponse (*5*). Weighted prevalence estimates describing the sociodemographic, behavioral, and clinical characteristics of AI/AN patients with HIV infection in care were calculated with accompanying 95% confidence intervals (CIs). Based on mental health results found in this descriptive analysis, mental health and peer group support services received and needed were also described.

AI/AN classification was determined by self-identified AI/AN race, regardless of ethnicity or other racial group identity (*2*). Poverty was defined as income at or below the HHS annual poverty guidelines.* Depression was defined as self-reported symptoms consistent with a diagnosis of major/other depression in the past 2 weeks based on the Patient Health Questionnaire-8 (PHQ-8) scale with major/other depression defined as a PHQ-8 score ≥2. Binge drinking was defined as consumption of four or more (females) or five or more (males) alcoholic drinks in one sitting in the past 30 days. Antiretroviral therapy (ART) adherence was defined as taking all prescribed HIV medicines in the past 3 days. Sustained viral suppression was defined as <200 copies of viral RNA/mL in all viral load tests during the past 12 months. Need for support services was defined as needing, but not receiving, mental health or HIV peer group support services during the past 12 months.

AI/AN patients (666) accounted for 3.6% (95% CI = 3.1–4.1) of the MMP population. Among AI/AN patients with HIV infection, 65% identified as being part of more than one racial group, and 29% identified as Hispanic/Latino ethnicity (Table 1). Fifty-one percent had household incomes at or below the HHS poverty guidelines, 12% experienced homelessness in the past 12 months, and 6% had been incarcerated in the past 12 months. Internalized HIV-related stigma^†^ was reported by 78% of patients, and 37% experienced health care discrimination since testing positive for HIV.^§^

**TABLE 1:** Sociodemographic characteristics of American Indian/Alaska Native (AI/AN)* adults living with human immunodeficiency virus (HIV) infection receiving medical care (N = 666) — Medical Monitoring Project, United States, 2011–2015

Characteristic	Total	%^†^ (95% CI^§^)
**Race**		
Single-race AI/AN	249	36 (31–40)
Multiple-race AI/AN	417	65 (60–69)
**Ethnicity**		
AI/AN, non-Hispanic/Latino	455	71 (65–77)
AI/AN, Hispanic/Latino	211	29 (23–35)
**Gender**		
Male	491	74 (69–78)
Female	157	23 (20–27)
Transgender^¶^	18	3 (2–5)
**Age group (yrs)**		
18–29	45	7 (5–9)
30–39	106	16 (14–19)
40–49	230	34 (30–37)
≥50	285	43 (40–47)
**Sexual identity**		
Homosexual	279	43 (39–48)
Heterosexual	306	46 (41–52)
Bisexual	67	10 (8–13)
**Education**		
<High school	172	25 (22–29)
High school/General educational development certificate	163	23 (20–26)
>High school	331	51 (47–56)
Household income at/below poverty in past 12 months**		
Yes	334	51 (46–55)
No	308	50 (45–54)
**Homeless in past 12 months^††^**		
Yes	78	12 (9–14)
No	588	88 (86–91)
**Jail in past 12 months**		
Yes	40	6 (4–8)
No	626	94 (92–96)
**Health insurance in past 12 months**		
Private only	141	22 (19–26)
Any public	445	65 (61–70)
Only Ryan White coverage or uninsured	76	12 (9–16)
**Any HIV-related stigma^§§^**		
Yes	519	78 (74–81)
No	140	22 (19–26)
**Any health care discrimination** ^¶¶^		
Yes	241	37 (32–42)
No	417	63 (58–68)

**Abbreviation:** CI = confidence interval.

* Self-identified AI/AN race, regardless of ethnicity or other racial groups.

^†^ Percentages are weighted percentages and might not sum to 100% because of rounding.

^§^ 95% CIs incorporate weighted percentages

^¶^ Patients were classified as transgender if sex at birth and gender reported by patient were different, or if patient’s gender identity was transgender.

** Poverty guidelines as defined by the U.S. Department of Health and Human Services. https://aspe.hhs.gov/poverty/faq.cfm.

^††^ Living on the street, in a shelter, in a single-room-occupancy hotel, or in a car.

^§§^ Agreed with any of the following statements: it is difficult to tell people about my HIV infection, being HIV-positive makes me feel dirty, I feel guilty that I am HIV positive, I am ashamed that I am HIV-positive, I sometimes feel worthless because I am HIV-positive, and I hide my HIV status from others.

^¶¶^ Health care discrimination was defined as a health care worker exhibiting hostility or lack of respect, giving the patient less attention than others, or refusing the patient service since the patient tested positive for HIV.

Among AI/AN patients with HIV infection in care, 27% had symptoms consistent with major/other depression in the past 2 weeks, 12% were dissatisfied with their social support, 20% reported binge drinking, 32% used noninjection drugs in the past 12 months, 5% injected drugs in the past 12 months, and 46% currently smoked cigarettes (Table 2). Eight percent of AI/AN patients with HIV infection had condomless sex with a partner who had a negative or unknown HIV status while the patient was not sustainably virally suppressed during the past 12 months. In terms of clinical characteristics, 86% of AI/AN patients on ART were adherent, 64% had achieved sustained viral suppression, and 76% had achieved viral suppression as of their most recent viral load test in the past 12 months.

**TABLE 2:** Behavioral and clinical characteristics of American Indian/Alaska Native (AI/AN)* adults living with human immunodeficiency virus (HIV) infection receiving medical care (N = 666) — Medical Monitoring Project, United States, 2011–2015

Characteristic	Total	%^†^ (95% CI^§^)
**Depression in past 2 weeks**		
Major/Other depression	169	27 (23–30)
None	484	73 (70–77)
**Satisfied with social support**		
Very dissatisfied	42	6 (5–8)
Somewhat dissatisfied	39	6 (4–8)
Somewhat satisfied	141	24 (20–28)
Very satisfied	382	64 (60–67)
**Binge drinking in past 30 days^¶^**		
Yes	132	20 (17–23)
No	524	80 (77–83)
**Any noninjection drugs in past 12 months**		
Yes	216	32 (27–35)
No	445	68 (64–73)
**Injected drugs in past 12 months**		
Yes	35	5 (3–7)
No	625	95 (93–97)
**Currently smokes cigarettes**		
Yes	302	46 (42–50)
No	359	55 (50–58)
**Sex without a condom with partner with HIV-negative or unknown status in past 12 months**		
Yes	87	14 (11–17)
No	540	86 (83–89)
**Sex without a condom with HIV-negative or unknown status partners while not sustainably virally suppressed in past 12 months**		
Yes	49	8 (5–10)
No	578	92 (90–95)
ART adherence**		
100% adherent	519	86 (83–90)
Not 100% adherent	87	14 (11–17)
**Sustained viral suppression in past 12 months**		
Yes	432	64 (60–68)
No	234	36 (32–41)
**Most recent viral load suppressed in past 12 months**		
Yes	512	76 (73–80)
No	154	24 (20–27)

**Abbreviations:** ART = antiretroviral therapy; CI = confidence interval.

* Self-identified AI/AN race, regardless of ethnicity or other racial groups.

^†^ Percentages are weighted percentages and might not sum to 100% because of rounding.

^§^ 95% CIs incorporate weighted percentages.

^¶^ Consumption of four or more (females) or five or more (males) alcoholic drinks in one sitting in the past 30 days.

** Taking all prescribed HIV medicines in the past 3 days.

Peer support group services were received by 17% of AI/AN patients, whereas 11% needed but did not receive these services (Figure). Approximately one third of AI/AN patients received mental health services, and 8% needed but did not receive mental health services.

**FIGURE:**
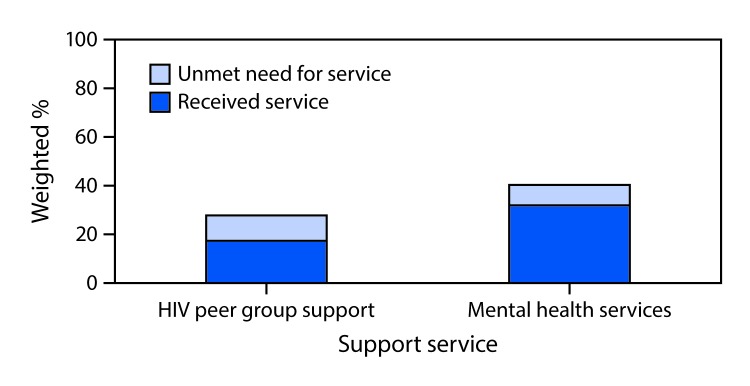
Mental health and peer group support service needs* among American Indian/Alaska Native (AI/AN)^†^ adults receiving human immunodeficiency virus (HIV) care (N = 666) — Medical Monitoring Project, United States, 2011–2015 * Need was defined as needing, but not receiving mental health or HIV peer group support services during the past 12 months. ^†^ Self-identified AI/AN race, regardless of ethnicity or other racial groups.

## Discussion

In this analysis, levels of viral suppression among AI/AN patients with HIV infection in care were suboptimal. Moreover, compared with other racial/ethnic groups, AI/AN patients have a higher rate of poverty, which is associated with poor physical and mental health outcomes (*1*). The prevalence of major/other depression (27%) among AI/AN patients in HIV care was similar to that among all adult patients in HIV care (25%) (*6*). The prevalence of stigma (78%) among AI/AN patients in HIV care, although high, was also similar to stigma among all adult patients in HIV care (79%) (*7*). The high prevalence of poverty, depression, stigma, and alcohol use in this population might be caused in part by racial and historical inequities and is not intrinsic to AI/AN cultures (*2*). Receiving culturally appropriate mental health and peer group support services could reduce symptoms of depression and increase social support (*8*); in this analysis, some AI/AN patients with HIV infection needed but did not receive these services.

Despite factors such as poverty and depression, which are often associated with suboptimal achievement of viral suppression, the prevalence of viral suppression among AI/AN patients in HIV care was similar to or higher than that among other racial/ethnic groups. AI/AN patients had prevalences of sustained viral suppression that were similar to those among white patients (66%) and higher than those among black (49%) and Hispanic/Latino (59%) patients in HIV care (*9*). However, the prevalence of viral suppression among AI/AN patients was lower than the national prevention goal of 80% for persons with diagnosed HIV infection.

The findings in this report are subject to at least three limitations. First, analysts pooled multiple years of data and could not analyze trends over time because of the small sample size of AI/AN patients in each MMP cycle year. Second, although the MMP sampling design was intended to represent all adult patients with HIV infection in outpatient settings in the United States, it did not include Indian Health Services (IHS) facilities, tribal lands, or some areas with a high concentration of AI/AN persons; however, the majority of AI/AN persons do not live on tribal lands (*10*). Finally, interview data were obtained by self-report, which might be susceptible to recall or social desirability biases.

From 2011 to 2016, diagnoses of HIV infection among AI/AN patients increased by 70% (*1*). CDC is currently working with IHS and tribal leaders to implement effective, scalable prevention approaches to support AI/AN patients. In light of the fact that almost 80% of AI/AN patients with HIV infection reported experiencing stigma related to their HIV status, and that more than a third reported experiencing discrimination in health care settings, it is evident that culturally appropriate HIV education, interventions, and care remain priorities (*2*). CDC provides culturally competent capacity-building assistance to IHS prevention programs, such as the Project Red Talon,^¶^ which works to achieve a more coordinated national and Northwest tribal response to HIV. Community-based interventions, such as CDC’s Let’s Stop HIV Together** media campaign might also help to reduce HIV-related stigma (*7*).

Because of historical factors affecting AI/AN populations, AI/AN patients receiving HIV care face unique circumstances that might interfere with their ability to achieve sustained viral suppression, including a high prevalence of poverty, depression, stigma, and substance use. It is important that HIV providers and clinics screen for these issues and offer referrals to mental health services and HIV peer group support as appropriate. Many community-based and tribal organizations are positioned to help AI/AN populations access culturally appropriate HIV and ancillary services to improve their health outcomes and reduce HIV-related health disparities.

**Summary**

**What is already known about this topic?**

In 2016, American Indians/Alaska Natives (AI/ANs) had the fourth highest human immunodeficiency virus (HIV) infection diagnosis rate among all racial/ethnic groups. During 2011–2016, diagnoses of HIV infection among AI/AN patients increased by 70%. Little has been published about characteristics of AI/AN patients with HIV infection.

**What is added by this report?**

Among adults receiving HIV care from 2011 to 2015, AI/AN patients had high poverty levels (51%), depression (27%), HIV stigma (78%), and suboptimal sustained HIV viral suppression (64%).

**What are the implications for public health practice?**

Providers serving AI/AN patients should offer screening and referrals for mental health and peer support services to improve the health of this population and help them achieve viral suppression.

## Footnotes

* Poverty guidelines as defined by the U.S. Department of Health and Human Services. https://aspe.hhs.gov/poverty/faq.cfm.

† Any internalized HIV-related stigma was defined as agreeing with any of the following statements from the Internalized AIDS-Related Stigma Scale: it is difficult to tell people about my HIV infection, being HIV-positive makes me feel dirty, I feel guilty that I am HIV-positive, I am ashamed that I am HIV-positive, I sometimes feel worthless because I am HIV-positive, and I hide my HIV status from others.

§ Health care discrimination was defined as a health care worker exhibiting hostility or lack of respect, giving the patient less attention than other patients, or refusing the patient service since the patient tested positive for HIV.

¶
https://npin.cdc.gov/featured-partner/project-red-talon .

**
https://www.cdc.gov/actagainstaids/campaigns/lsht/ .
